# Beyond emergency relief: The role of U.S. foreign health assistance amid growing displacement and climate change

**DOI:** 10.1371/journal.pgph.0005321

**Published:** 2025-11-03

**Authors:** Timothy H. Holtz, Lisa Hilmi, Maya M. Rao, John Borrazzo, Dennis Cherian, Colleen K. GallagherThomas, Mark Hetfield, Dennis J. King, Barry S. Levy, Jed Meline, Marie D. Price, John P. Quattrochi, Adam K. Richards, Lynn R. Goldman, Steven J. Hansch

**Affiliations:** 1 Sumner M. Redstone Global Center for Prevention and Wellness, The George Washington University Milken Institute School of Public Health, Washington, D.C., United States of America; 2 CORE Group, Washington, D.C., United States of America; 3 The George Washington University Milken Institute School of Public Health, Washington, D.C., United States of America; 4 Corus International, Washington, D.C., United States of America; 5 Relief International, Washington, D.C., United States of America; 6 HIAS, Silver Spring, Maryland, United States of America; 7 Retired from U.S. Government, Washington, D.C., United States of America; 8 United Nations Office for Coordination of Humanitarian Affairs (OCHA), New York, New York, United States of America; 9 Department of Public Health and Community Medicine, Tufts University School of Medicine, Boston, Massachusetts, United States of America; 10 Project Hope, Washington, D.C., United States of America; 11 Department of Geography and Environment, George Washington University, Washington, D.C., United States of America; 12 Department of Global Health, Georgetown University, Washington, D.C., United States of America; 13 ASEAN Institute for Health Development, Mahidol University, Bangkok, Thailand; 14 World Hunger Education Service, Washington, D.C., United States of America; PLOS: Public Library of Science, UNITED STATES OF AMERICA

## 1. Introduction

Global displacement, both forced and environmentally induced, is on the rise, with intensifying health consequences [1,2]. Global health assistance for people impacted by displacement and climate hazards is a humanitarian priority, critical to averting deaths and promoting safety and security both domestically and abroad. The global humanitarian aid system is rapidly losing technical capacity amid the decline of U.S. foreign assistance. The U.S. has historically spearheaded best practices, and evidence-based approaches in global health, humanitarian assistance, and disaster risk reduction (DRR). However, its credibility in the foreign aid system is deteriorating due to drastic reductions in programs and staff at U.S. Department of State (DOS), former U.S. Agency for International Development (USAID), and the Department of Health and Human Services. Given this context, the recent decline in U.S. global health funding is gravely concerning.

The U.S. should preserve technical capacity in global health, DRR, and humanitarian assistance and enhance coordination with other international actors during the transition of aid programs to DOS. Broadly eliminating technical positions and structures risks the loss of important institutional knowledge. Historically the largest contributor to foreign assistance, the U.S. must now refocus to complement other actors with its unique technical expertise. Continued U.S. engagement in these global efforts is important for the reasons noted in the text box.

**Box 1. Reasons why U.S. foreign assistance for health and climate adaptation should continue.**

- The U.S. has been uniquely positioned to develop innovative solutions for health assistance in displacement crises, given its proven capacity to collaborate and provide technical expertise.
- Investments in disaster risk reduction (DRR) can mitigate costly climate-related health hazards. Every $1 invested in adaptation efforts can yield over $10.50 in economic, social and environmental benefits over a 10-year period [3].
- Investments in preventive health programs, like the 2017 measles vaccination campaign that averted 77,000 cases in Rohingya refugee camps, are highly efficient [4]. Greater health benefits are achieved on fewer dollars spent, particularly when the resources and talents of local communities are engaged. Even modest U.S. contributions to such programs can also amplify the efforts of other foreign assistance actors, like United Nations agencies and the European Union.

With the health consequences of displacement and climate change of urgent concern, coordinated U.S. foreign assistance is critical to avert deaths and promote global health security.

## 2. Key issues and recommendations

1. A record 123 million people are displaced globally, and this trend is projected to continue [2]. Social, political, economic, and environmental factors contribute to displacement and its associated health risks [5]. The largest and most lethal humanitarian and displacement crises, such as wars and civil conflicts, are often protracted, while humanitarian assistance often prioritizes short-term responses, which are inadequate to address real health needs [6].
  U.S. foreign assistance should extend beyond emergency response to include adaptation efforts, given the increasing frequency and duration of displacement and humanitarian crises. With technical support in DRR, at-risk communities can better adapt to climate-related health hazards and enhance their capacity to remain in places where they have strong ties [7]. For example, the SERVIR multi-hazard early warning system in Nepal successfully strengthened local capacity to anticipate climate hazards and develop evidence-based adaptation plans [8].
2. Climate hazards, such as drought, flooding and heat waves, are increasing in severity and frequency and a threat multiplier of displacement [9]. Conflict can contribute to displacement and be influenced by climate change due to tensions over diminishing natural resources [10].
  U.S. foreign health assistance should be coordinated with global actors and communities to address the complex interplay of environmental, political, and economic drivers of displacement and enable communities to drive these efforts. The U.S. is well positioned to address these challenges with holistic approaches, given its expertise in data science, technological innovation, and health information systems. One example is the International Rescue Committee’s collaboration with Gavi, the Vaccine Alliance, and local partners in the Horn of Africa on securing access to routine immunization in conflict- and flood-prone areas, leveraging a data-driven protocol to map accessible routes for critical health service delivery [11].
3. Mass forced and environmentally induced displacement increases risk of communicable and non-communicable diseases, injuries, and maternal and mental health challenges [10]. Climate change also alters how diseases spread, particularly in displaced communities exposed to crowded conditions, limited mobility and constrained healthcare access [10].
  The U.S. should further invest in cross-cutting, technologic innovations, such as last-mile supply chain enhancements, telemedicine and mobile health records, that can address multiple access constraints during displacement through efficient and far-reaching delivery of medicine, clean water and skilled healthcare provision. One example of successful cross-sector collaboration is HERA Digital Health’s partnership with Cloudfare to implement a mobile healthcare application that helps displaced people identify the nearest health center and maintain their medical records [12].
4. Disrupted services and disease outbreaks compounded by climate change can threaten global health security, putting people at risk of health hazards like measles, cholera, and dengue, both in countries where they occur as well as across borders. These challenges will continue to persist in areas where local government, civil society, and the private sector have limited capacity to provide infrastructure and basic services.
  U.S. funding should be quickly and directly accessible to local civil society and faith-based organizations—who have close ties to affected communities and are first-line responders—as well as local government structures to ensure lasting impact. Decision-making power should shift from donors and Global North actors to local actors, which could include expansion of locally led pooled funds and enhanced coordination through groups like the Network for Empowered Aid Response [13].
5. Across LMICs, the health-related costs of climate change are projected to exceed $20.8 trillion by 2050 [14]. These losses will be disproportionately felt among people living in poverty or lacking educational opportunities.
  Given the cost effectiveness and high return on investment of hazard mitigation efforts in LMICs, the U.S. should scale up technical support, coordination and investment in DRR and adaptation interventions. While the DOS should play a key role, DRR financing for LMICs could also be channeled through the Development Finance Corporation, the World Bank Group or regional development banks.
6. Safe access for health care delivery and recognition of humanitarian principles is rapidly deteriorating, for example, in Gaza and Sudan. Attacks on healthcare facilities and personnel increased by 15% in 2024 compared to 2023 [15]. These violations of humanitarian law have profound impacts, driving cross-border displacement and reducing access to healthcare in insecure areas.
  U.S. foreign assistance should support international efforts that safeguard healthcare, public health, and humanitarian personnel in conflict and displacement contexts. The U.S. can further support international and local aid organizations through advocacy for humanitarian corridors, removal of bureaucratic impediments, and addressing violations of international humanitarian law.

## 3. Conclusion

U.S. global health assistance is an urgent humanitarian priority, particularly amid rapidly growing displacement and climate-induced crises. Continued deprioritization will ultimately result in lives lost. Critical to averting deaths and promoting global health security, U.S. technical capacity in global health should be reinvigorated through synergies with the private sector, international actors, and local institutions. When engaging with government stakeholders on this issue during this challenging climate, global health advocates should highlight the need for sufficient resources and cross-sector collaboration to implement cost-effective interventions that lessen the health impacts of climate hazards and displacement.
